# Innate Immunity to *Leishmania* Infection: Within Phagocytes

**DOI:** 10.1155/2014/754965

**Published:** 2014-07-07

**Authors:** Marcela Freitas Lopes, Ana Caroline Costa-da-Silva, George Alexandre DosReis

**Affiliations:** Carlos Chagas Filho Institute of Biophysics, Federal University of Rio de Janeiro, 21941-900 Rio de Janeiro, RJ, Brazil

## Abstract

Infection by *Leishmania* takes place in the context of inflammation and tissue repair. Besides tissue resident macrophages, inflammatory macrophages and neutrophils are recruited to the infection site and serve both as host cells and as effectors against infection. Recent studies suggest additional important roles for monocytes and dendritic cells. This paper addresses recent experimental findings regarding the regulation of *Leishmania major* infection by these major phagocyte populations. In addition, the role of IL-4 on dendritic cells and monocytes is discussed.

## 1. Introduction

Infection of humans with* Leishmania* surmounts 1.3 million new cases each year. Parasites infect and survive within phagolysosomal vesicles in host macrophages. Following infection with* Leishmania*, macrophages produce reactive oxygen species (ROS), cytokines, and chemokines and recruit an early inflammatory reaction [1, 2]. Interactions with inflammatory neutrophils either increase or decrease* L. major* replication in macrophages depending on host genotype and through mechanisms involving TGF-*β* or neutrophil elastase [3–5]. Recent studies suggest that additional phagocytes such as monocytes and dendritic cells (DCs) play important roles in infection, both as host and as effector cells. Here we discuss recent experimental findings regarding regulation of* L. major* infection by these major phagocyte populations. In addition, the role of IL-4 on DC and monocyte responses to infection is discussed.

## 2. Macrophage Activation

In response to microbial stimulation, macrophages differentiate into distinct M1 and M2 phenotypes [6, 7]. Both M1 and M2 macrophages are induced in the course of* Leishmania* infection. Microbial stimulation following priming with the Th1 cytokine IFN-*γ* leads to classically activated or M1-type macrophages [6]. M1 macrophages express nitric oxide (NO)-dependent leishmanicidal activity and are important for control of* Leishmania* infection [7]. By contrast, M2-type macrophages, induced by the Th2 cytokine IL-4, express arginase and play an important role in tissue repair [6]. Expression of PPAR-*γ* is also required for induction of M2 macrophages [8]. Mice lacking M2 or alternatively activated macrophages due to genetic deficiency of either the IL-4 receptor or PPAR-*γ* are more resistant to* L. major* infection [8, 9]. In addition, host IgG promotes infection by* Leishmania* due to early effects on macrophage differentiation [10]. Ligation of Fc*γ* receptors by IgG immune complexes induces IL-10 production and imprints a regulatory or M2b phenotype in macrophages, which is permissive for* Leishmania* replication [7, 11]. These results suggest that* L. major* takes advantage of M2 macrophages to replicate in the host.

Infection by* L. major* takes place in the context of inflammation and tissue repair induced by the insect bite [12–14]. Insect salivary molecules play an important role in the establishment of infection. Maxadilan, an insect derived salivary peptide, modulates the immune response of the host and reprograms dendritic cell maturation to facilitate infection [15, 16]. In addition, molecular and cellular elements recruited by the inflammatory reaction modulate macrophage/*Leishmania* interactions. Tissue repair is a conserved response to injury characterized by an initial influx of neutrophils, followed by monocyte/macrophages and fibroblasts. Repair cannot be completed until inflammation is resolved, and, in the case of* L. major* infection, chronic ulcers are associated with persisting infiltrates of neutrophils [13]. Dynamic intravital microscopy of* L. major* infection site indicates that, after 1 day of infection, parasites localize mainly inside neutrophils [14]. Later, neutrophils are cleared and parasites become localized to monocyte/macrophages [14]. Interestingly, viable parasites are released from apoptotic neutrophils in the vicinity of macrophages [14]. Therefore, it is possible that* Leishmania* hides in neutrophils until apoptosis forces the parasites to infect a macrophage.

## 3. Early Macrophage Responses following Infection with* L. major*

Infection of resident macrophages plays an important sentinel role in early stages of* Leishmania* infection. Macrophages respond to infectious stress by either adapting or undergoing apoptosis. We investigated the role of cellular stress and apoptosis in macrophages infected with* L. major* in both susceptible and resistant mice. FasL plays a major role in early responses of susceptible BALB/c mice to infection. Infection induces FasL-dependent apoptosis of Mac-1/CD11b^hi^ resident macrophages, concomitant with secretion of chemokines KC and MIP-1*α*, and neutrophil extravasation [4]. Apoptosis and chemokine secretion induced by* L. major* in resident macrophages can be prevented with a neutralizing antibody specific for FasL, and neutrophil extravasation is reduced in FasL-deficient* gld* mutant mice [4]. These results agree with studies showing resident macrophage demise, chemokine secretion, and neutrophil extravasation following FasL stimulation of macrophages [17].

In contrast, early responses of resistant mice to infection are independent of FasL. Macrophages from B6 mice do not undergo apoptosis upon* L. major* infection. Chemokine secretion is independent of FasL expression, and neutrophil extravasation induced by infection is preserved in FasL-deficient mice [18]. In addition, we found no sign of macrophage death, and infected B6 macrophages remained viable as judged by constitutive secretion of lysozyme [18]. The reason for the different role of FasL in these mouse strains is unknown. However, BALB/c and B6 mice express a genetic polymorphism in FasL that affects biological activity, and B6 FasL has less cytotoxic activity than BALB FasL [19].

Although infection with* Leishmania* fails to induce apoptosis, it induces a cellular stress response in resident B6 macrophages characterized by increased production of ROS, activation of the stress activated protein kinases JNK, activation of c-Jun, and increased expression of FasL in resident macrophages [18]. Infection increased secretion of cytokines/chemokines TNF-*α*, IL-6, TIMP-1, IL-1RA, G-CSF, TREM, KC, MIP-1*α*, MIP-1*β*, MCP-1, and MIP-2 in resident macrophages. Secretion of KC is blocked either by addition of antioxidants or by a JNK inhibitor, suggesting that the stress response is involved in chemokine secretion. Interestingly, antioxidants and JNK inhibitor also blocked the intracellular growth of parasites [18], although the mechanisms involved remain to be determined. These results suggest that a cellular stress response by resident B6 macrophages recruits inflammatory cells but also promotes the intracellular survival/growth of the parasite [18].

## 4. Infection with* L. major* Interferes with Cytokine-Induced Macrophage Differentiation

**Figure 1:**
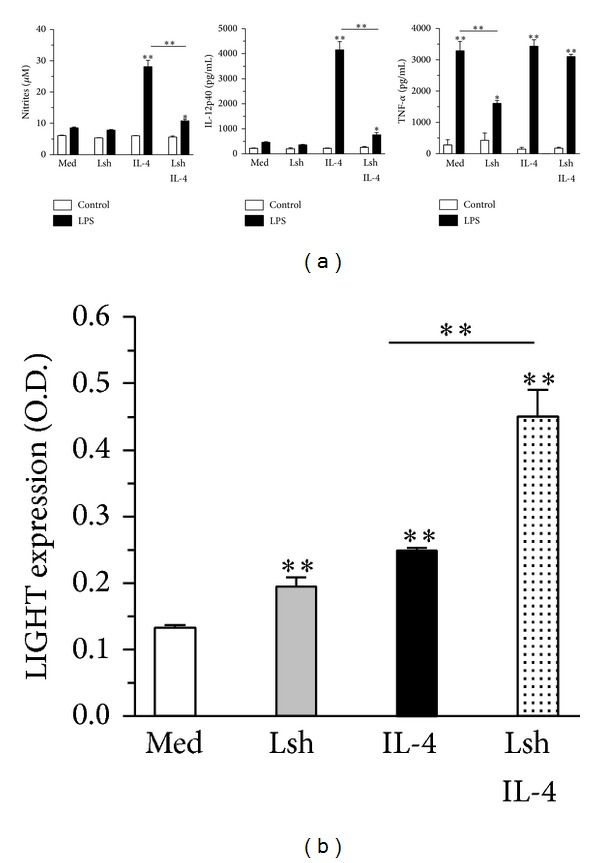
Infection with* L. major* reduces production of NO and IL-12 and increases expression of M2 marker LIGHT by differentiated macrophages. (a-b) Bone marrow derived macrophages (BMDM from B6 mice) were infected or not with* L. major* after 6 days. Next day, all BMDM were treated with IFN-*γ*, and next day, some of the BMDM cultures were treated with IL-4. (a) After 3 d, cells were washed and restimulated with medium (control) or LPS. After 2 additional days, the levels of nitrites, IL-12p40, and TNF-*α* were determined. (b) After 3 d, expression of LIGHT was evaluated by cellular ELISA. Results are expressed as mean and SE of triplicates (a) or quadruplicates (b), *P < 0.05, **P < 0.01.

In response to cytokines, macrophages differentiate to effector functions. Macrophages treated with a combination of IFN-*γ* plus IL-4 express leishmanicidal activity [20] and, following restimulation with LPS, secrete nitrites and IL-12, two markers of M1 differentiation [21]. We investigated the effect of prior* Leishmania* infection on cytokine induced macrophage differentiation. Macrophages treated with IFN-*γ* plus IL-4 produced increased levels of NO and IL-12p40 following restimulation with LPS (Figure 1(a)). Prior infection with* L. major *substantially reduced the ability to produce NO and IL-12p40 (Figure 1(a)). Prior infection did not reduce TNF-*α* response (Figure 1(a)), arguing against a defect in the response to LPS and suggesting a specific defect in IL-12 and nitrite secretion. We also investigated expression of LIGHT, a marker of M2b macrophage differentiation [7]. Prior infection with* L. major* increased expression of LIGHT in macrophages treated with IFN-*γ* plus IL-4 (Figure 1(b)). Therefore, prior infection with* L. major* reduces expression of M1 differentiation markers, whereas it increases expression of M2 differentiation marker. Interestingly, infection with* L. major* after treatment with IFN-*γ* did not modulate expression of differentiation markers (results not shown). Together, these data suggest that* L. major* modulates macrophage differentiation to ensure its intracellular survival. Interestingly, production of ROS is required for inducing M2, but not M1 macrophage differentiation [22], suggesting that the initial stress response could be involved.

## 5. Engulfment of Neutrophils Regulates Infection of Macrophages

Neutrophils play important roles in the innate immune response. Neutrophils are short lived cells that undergo spontaneous apoptosis following transmigration of blood vessels [23]. Neutrophil apoptosis induces phagocytic clearance and secretion of cytokines by macrophages, which are important for resolution of inflammation and tissue repair [23]. Interestingly, different outcomes of* L. major* infection and cytokine production are observed in macrophages engulfing apoptotic neutrophils, depending on host genetic background [3]. Engulfment of apoptotic BALB/c neutrophils induces production of TGF-*β*, but not TNF-*α* in macrophages, and increases growth of* L. major *in a manner dependent on PGE_2_ and TGF-*β* [3]. Depletion of neutrophils with anti-Gr1 antibody reduces, and adoptive transfer of apoptotic neutrophils increases infection in lymph nodes of BALB/c mice, suggesting a disease promoting role of neutrophils [3]. On the other hand, engulfment of B6 neutrophils induces production of TNF-*α*, and not TGF-*β*, in infected macrophages and reduces* L. major* intramacrophagic load in a manner dependent on TNF-*α* [3]. Depletion of neutrophils with anti-Gr1 antibody increases, and adoptive transfer of apoptotic neutrophils decreases infection in lymph nodes of B6 mice, role. Neutralization of neutrophil elastase (NE) with a specific inhibitor peptide abrogates the inflammatory clearance of B6 neutrophils and increases infection in lymph nodes [3].

## 6. Macrophage Activation and Differentiation Induced by Neutrophil Engulfment and Neutrophil Elastase

Clearance of apoptotic cells can be proinflammatory in the presence of additional innate immune stimuli, such as TLR ligands [24, 25]. This observation helps to explain the puzzling proinflammatory effects of phagocytosis of apoptotic B6 neutrophils. NE triggers proinflammatory responses through TLR4 [26, 27]. Purified NE triggers TNF-*α* production by macrophages and induces leishmanicidal activity in a manner dependent on TLR4 [5]. Interestingly, B6 neutrophils release 2-3-fold more NE than BALB/c neutrophils. In addition, mutant* pallid* B6 neutrophils, which fail to release NE, do not induce killing of* L. major* [5]. These results suggest that the proinflammatory and leishmanicidal activities of B6 neutrophils are due to efficient amounts of released NE and perhaps other released granule proteins.

One important question is whether neutrophil engulfment imprints a particular phenotype in macrophages. Our results indicate that proinflammatory, but not antiinflammatory clearance of neutrophils induces a sustained regulatory or M2b phenotype in macrophages, characterized by low IL-12p70 and high IL-10 production following restimulation with LPS, increased expression of LIGHT, induction of Th2 responses, and permissive replication of* L. major* [21]. As expected, the ability of senescent neutrophils to induce the regulatory phenotype requires NE activity and TLR4 expression [21]. Moreover, previous injection of senescent neutrophils enhances subsequent infection* in vivo* [21]. These results suggest that induction of regulatory macrophages plays a permissive role in establishment of infection.

## 7. Monocytes and DCs as Phagocytes and Effector Cells

Monocytes are crucial to immunity against* L. major* infection as precursors of macrophages and inflammatory DCs. On the other hand, the role of monocytes as key effectors of parasite killing remains elusive, since current monocyte depletion models fail to spare the other cell types. Accordingly, CCR2 is required for mobilization of monocytes from bone marrow [28] and for effective control of* L. major* infection [29–31]. Whether CCR2-dependent immunity to* L. major* relies on direct effector role of monocytes, in the generation of effector macrophages/DCs, or both is still debatable [29–32]. Conversely, attempts to deplete only neutrophils by using antibody against Gr1, which encompasses both Ly6C (monocyte) and Ly6G (neutrophil) epitopes, more likely result in monocyte depletion as well.

Infection with* L. major* increases both myelopoiesis and the number of circulating myeloid precursors [33]. In addition, purified blood monocytes are already able to phagocytose* L. major* parasites [34]. Blood monocytes, however, may not match the activation state of elicited monocytes in the infection site [34, 35]. More important, different degrees of monocyte maturation, either in the bone marrow or in the infection site, might confer susceptibility or resistance to* L. major* infection in BALB/c versus B6 mice [13, 36, 37].

Immature myeloid cells were once considered as “safe targets” for* Leishmania* infection [33, 38]. Indeed, immature macrophages expressing myeloid markers were found as major parasite reservoirs in skin lesions of BALB/c mice infected with* L. major* even 4 weeks after infection [13, 36, 38]. Recent evidences indicate that monocytes/macrophages predominate as infected cells at early (3 days) and late (4 wks) time points in skin lesions, whereas infected DCs accumulate at 4 weeks in lesions and are always dominant in B6 or BALB/c draining lymph nodes [32, 39]. In spite of differences in phagocyte populations in different sites and routes of infection [40], detailed analyses in B6 mice show that Ly6C^hi^ monocytes are the major infected cell at the ear infection site from 1 day to 1 week postinfection, remaining infected thereafter, whereas DCs/macrophages predominate as infected cells upon 2 weeks of infection [41]. Recent results also suggest that both monocytes and monocyte-derived dendritic cells (Mo-DCs) may either express effector activity against* Leishmania* parasites or contribute to infection in different models. For example, Mo-DCs became infected at the infection site and migrate to LN to induce Th1 responses [32]. By contrast, DCs that were infected through uptake of infected neutrophils fail as APCs for CD4 T cells [41].

Nonetheless, the effector functions of both monocytes and DCs were recently evidenced in the* L. major* model (Table 1). Following* L. major* infection and platelet activation, Gr1^+^ monocytes from B6 mice are recruited to infection site through the CCR2 receptor [31]. Phagocytosis of* L. major *by monocytes ensues, followed by killing/disappearance of* L. major* parasites both* in vitro* and* in vivo* [31]. Although other mechanisms were not addressed, monocytes eliminated* L. major* infection in a Phox-dependent manner* in vitro* [31]. Ly6C^hi^ monocytes elicited by* L. major* infection can also kill parasites* in vitro* by producing NO [42]. A possible explanation to reconcile these apparently discrepant results is that peroxynitrites derived from both NO and ROS are involved as a more effective killing mechanism [43]. Injection of purified monocytes at the time of infection also helped infected B6 mice to control skin lesions and* L. major* parasites [42]. Treatment with all-*trans*-retinoic acid (ATRA) to induce monocyte maturation into macrophages releases T cell proliferation from suppression mediated by NO-producing immature monocytes [42]. However, treatment with ATRA also reduces parasite killing* in vitro* and promotes infection* in vivo* [42]. These results indicate that monocytes from B6 mice are authentic effector cells against* L. major* infection and that failure to recruit monocytes in CCR2 deficient mice has a major contribution to susceptibility to infection [31, 42]. Resistance to* L. major* infection has also been attributed to monocyte derived INOS-producing inflammatory DCs [30, 39]. However, activated monocytes share many markers with inflammatory DCs, including CD11c [44], and current markers fail to achieve a clear distinction between these phenotypes in the infection site [32]. Although CD11c has been considered as a key marker for DCs, a recent investigation on depletion of cells in a CD11c-DTR model indicates that monocytes are also depleted and that conclusions drawn from these models should be interpreted with caution [44]. In any case, DCs predominate in lymph nodes where they help immunity both as NO-producing effector cells [30, 39] and APCs to induce Th1 responses in* L. major* infection [32].

## 8. Protective Effects of IL-4 in* L. major* Infection

New evidence has challenged the role of IL-4 as the canonical Th2 cytokine that favors* L. major* infection. Early injection of IL-4 in susceptible mice promotes Th1 responses and resistance to* L. major* infection, through stimulation of DCs to produce IL-12 [45]. Similarly, in concert with TLR ligands, IL-4 and IL-4R*α* induce priming of Th1 responses by DCs [46]. Nonetheless, IL-4 also alternatively activates DCs in Th2 responses [46], in analogy to IL-4-alternatively activated macrophages [9]. Therefore, the role of IL-4 is target cell and context dependent. Interestingly, BALB/c mice deficient for IL-4R*α* expression on CD11c^+^ cells are highly susceptible to* L. major* infection [39]. These studies suggest that IL-4/IL-4R*α* signaling accounts for resistance in the acute phase of infection, by promoting DC-induced Th1 responses and classical macrophage activation [39]. As discussed here, however, IL-4R*α* deficiency in CD11c^+^ activated monocytes could also account for susceptibility to* L. major* infection. By contrast, defective IL-4R*α* expression in macrophages results in increased resistance to* L. major* by blocking alternative activation of macrophages [9].

Recent studies demonstrate that exacerbated Th2 responses help resistance to* L. major* in Th1-biased B6 model and that late treatment with anti-IL-4 (2–4 wks) increases susceptibility to infection [47]. Interestingly, NO-producing Gr1^+^ monocytes accumulate in the spleens of* L. major*-infected mice in this mixed Th1/Th2 model (unpublished results). Moreover, similar to findings with macrophages [9, 20, 48], monocytes elicited by* L. major* can be stimulated with a combination of IL-4 and IFN-*γ* to kill parasites in a NO-dependent manner [42]. IL-4 and IL-13, which share the same IL-4R*α*, induce myelomonopoiesis and therefore play a role both in the generation [49, 50] and the activation [51] of monocytes under certain conditions.

## 9. Concluding Remarks

**Figure 2:**
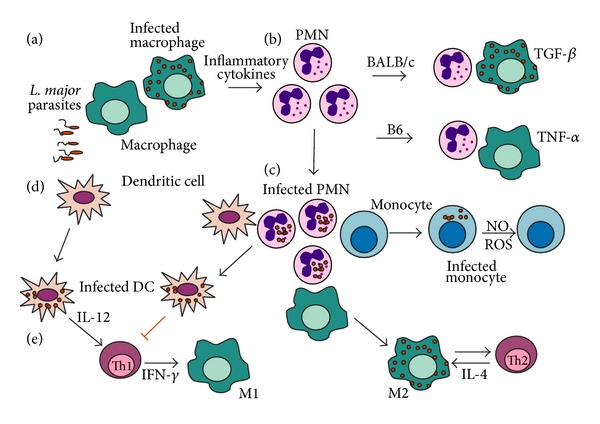
(a) Interactions among phagocytes in* L. major* infection. Upon* L. major* infection, tissue resident macrophages produce inflammatory cytokines and chemokines [4, 18] that recruit neutrophils and monocytes, which act as effector cells [31, 42] or give rise to inflammatory macrophages and DCs [30, 32]. (b) Neutrophils may either help or prevent parasite clearance by macrophages from B6 and BALB/c mice, respectively [3, 5]. (c) In addition, infected neutrophils help to propagate infection to macrophages, monocytes, and DCs [14, 41]. Moreover, macrophages primed by apoptotic neutrophils became permissive to subsequent infection and induce Th2 responses [21]. (d) DCs infected through efferocytosis of neutrophils fail to activate T lymphocytes [41]. Otherwise, infected DCs go to lymph nodes and induce Th1 responses [29, 32]. (e) Th1 cytokines activate M1 macrophages to kill parasites, whereas Th2 responses induce parasite-permissive M2 macrophages [6, 7].

Different phagocyte populations can be infected by* L. major* and express distinct responses that affect immunoregulation (Figure 2). Taken together, the studies discussed here also open new questions to the controversial role of IL-4/IL-4R*α* in* L. major* model [52]. Furthermore, monocytes and DCs can be relocated to the core of this issue, with direct implications for the development of new vaccines to Leishmaniasis [53].

## Figures and Tables

**Table 1:** The role of phagocytes and IL-4 in immunity to *L.  major* infection.

Phagocytes	Role in immunity	Experimental model	Outcome to infection	Ref.
Monocytes	APCs	Injected in B6 mice 4 wks upon infection	Generation of Mo-DCs/APCs (Th1 responses)	[32]
Monocytes	NO-producing effector cells	Injected in B6 mice upon infection	Resistance to acute infection	[42]
Monocytes	ROS-producing effector cells	B6-CCR2.KO	Susceptibility	[31]
DCs	APCs	B6-CCR2.KO	Susceptibility/Th2 response	[29]
DCs	APCs/NO-producing effector cells	B6-CCR2.KO	Defective recruitment of DCs to LN	[30]
DCs	APCs/NO-producing effector cells	CD11c^cre^IL-4R*α* ^flox/-^	Susceptibility/Th2 response	[39]
Macrophages	NO-producing effector cells	LysM^cre^IL-4R*α* ^flox/-^	Resistance to acute infection	[9]
